# A blinded, randomized and controlled multicenter clinical trial to assess the efficacy and safety of Leisguard^®^ as an immunotherapeutic treatment for healthy *Leishmania infantum*-seropositive dogs

**DOI:** 10.1186/s13071-023-05903-0

**Published:** 2023-10-04

**Authors:** Marta Baxarias, Giulia Donato, Cristina Mateu, Marta Salichs, Josep Homedes, Guadalupe Miró, Maria Grazia Pennisi, Laia Solano-Gallego

**Affiliations:** 1 Departament de Medicina i Cirurgia Animals, Facultat de Veterinària, Universitat Autònoma de Barcelona, Bellaterra, Spain; 2 Dipartimento di Scienze Veterinarie, Università di Messina - Polo Universitario Annunziata, Messina, Italy; 3 Ecuphar Veterinaria SLU, Barcelona, Spain; 4 Departamento de Sanidad Animal, Universidad Complutense de Madrid, Madrid, Spain

**Keywords:** Antibody, Canine, Domperidone, Interferon gamma, Leishmaniosis, PCR, Placebo

## Abstract

**Background:**

Domperidone (Leisguard^®^) is an immunomodulatory drug used as a preventive measure in healthy dogs. However, no studies have been published in healthy *Leishmania infantum*-seropositive dogs. The aim of this study was to evaluate the clinical efficacy and safety of domperidone as immunotherapy in *Leishmania*-seropositive healthy dogs.

**Methods:**

Sixty-seven dogs were treated with domperidone at 0.5 mg/kg and 44 dogs received placebo, once daily for 4 consecutive weeks. Monthly treatments were repeated every 4 months until the end of the 1-year follow-up period. Veterinary examinations were performed on days 0, 30, 120, 150, 240, 270 and 360. Samples of blood and urine were collected on days 0, 120, 240 and 360 for routine laboratory tests and quantitative in-house ELISA for the detection of *L. infantum*-specific antibodies. Furthermore, *Leishmania* real-time PCR and IFN-γ ELISA were performed at day 0 and the end of the study. Dogs that developed disease were withdrawn from the study and classified as sick dogs. Adverse drug reactions were reported.

**Results:**

Thirty dogs developed disease during the follow-up period: 13/67 (19.4%) in the group treated with domperidone and 17/44 (38.6%) in the placebo-treated group (*P* = 0.03). Low-seropositive dogs treated with domperidone (4/40, 9.1%) were significantly less likely to develop disease compared to low-seropositive dogs treated with placebo (7/24, 29.2%; *P* = 0.04), while no differences were found between domperidone (9/23, 39.1%) and placebo (10/20, 50%) in medium- to high-seropositive dogs. At the end of the study, a higher proportion of *Leishmania* PCR-positive dogs was observed in the placebo-treated group (16/33, 48.5%) compared to the domperidone group (13/51, 25.5%; *P* = 0.04). Furthermore, low-seropositive dogs treated with domperidone with an increase of IFN-γ concentration presented a higher increase than those treated with placebo at the end of the study. Four dogs treated with domperidone presented self-limiting diarrhea.

**Conclusions:**

Healthy dogs with low *L. infantum* antibody levels treated with domperidone were less likely to develop disease compared to placebo-treated dogs. Furthermore, domperidone presented a good safety profile.

**Graphical abstract:**

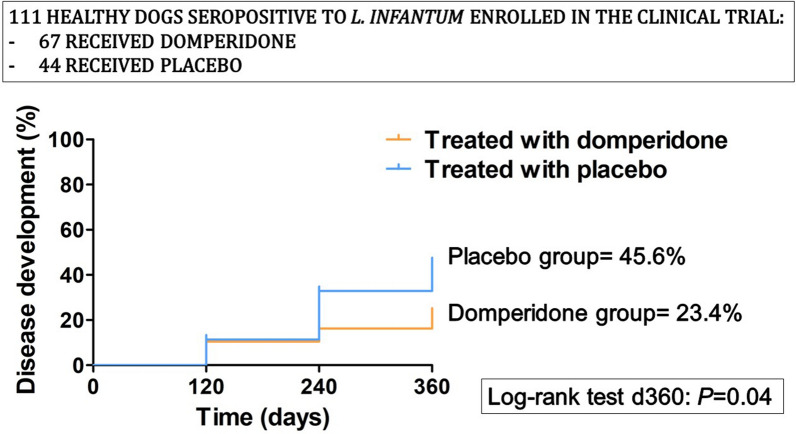

**Supplementary Information:**

The online version contains supplementary material available at 10.1186/s13071-023-05903-0.

## Background

Canine leishmaniosis (CanL) due to *Leishmania infantum* is prevalent in more than 80 countries worldwide [1, 2]. The disease is considered a major zoonosis in Europe, and its control and prevention constitute a major goal for veterinary and clinical health research and regulating agencies [3, 4].

A broad range of immune responses and clinical manifestations has been described in canine *L. infantum* infection [5, 6]. In fact, the development of clinical leishmaniosis is closely influenced by the immune response of the host, which is very complex, still fairly unknown and determined by not only by genetics but also acquired factors [7, 8]. The immune response requires a balance between inflammatory and regulatory responses to control *L. infantum* infection and avoid disease development [7, 8]. For example, a dog that displays a protective cell-mediated immune response characterized by interferon gamma (IFN-γ) release is able to stimulate the activation of macrophages to produce nitric oxide and reactive oxygen substances for intracellular killing of amastigotes. This process should be able to control *Leishmania* infection. In contrast, another dog that displays mainly a non-protective marked humoral immune response combined with absent or diminished cell-mediated immunity will be susceptible to *Leishmania* infection, present a high parasite burden and finally develop clinical disease [7]. Furthermore, as the infection progresses towards disease, there is a decrease of T cell proliferation and IFN-γ production and a lack of macrophage activation, resulting in a reduction of parasite elimination [8].

As the manifestations of leishmaniosis are closely influenced by the dog’s immune response, there is a wide and variable range of different clinical presentations. The most common clinical signs of CanL due to *L. infantum* are skin lesions, weight loss and generalized lymphadenomegaly, among a large variety of other clinical conditions [5, 9]. Furthermore, some laboratory findings such as hyperproteinemia, hyperglobulinemia, hypoalbuminemia, non-regenerative anemia and renal proteinuria are also suggestive of CanL [2, 5]. Four clinical stages of CanL have been designated based on clinical signs, clinicopathological abnormalities and serological status to classify dogs presenting clinical leishmaniosis, and different treatment protocols and prognoses are suggested for each clinical stage from stage I (mild disease) to stage IV (very severe disease) [10].

The treatment administered in CanL is usually long term, sometimes with no chance of discontinuation, and aims to reduce parasitic load [5]. Since there is no drug that can achieve a complete elimination of the parasite, a relapse of the disease would be expected [11]. The most common treatment consists on antimonials, which actively reduce parasitic load, together with allopurinol, which has a parasitostatic effect and, therefore, maintains parasitic load at low levels [2, 5, 11]. These drugs can cause significant adverse effects, most frequently nephrotoxicity [12, 13], urolithiasis and crystalluria [14, 15] or digestive disorders [16]. In addition, resistances to several of these drugs have also been documented such as resistances to antimonials [17] or allopurinol [18]. Considering the current knowledge that the immune system is the hallmark of the outcome of *Leishmania* infection and that the treatments used induce adverse effects and resistances, the most promising approach would be the use of immunotherapy to improve the specific immune response against parasites [19]. Immunotherapeutic products such as domperidone [20, 21] and dietary nucleotides and active hexose dietary compound (AHCC) [22] have been investigated in dogs with leishmaniosis.

Domperidone is a drug that has demonstrated positive results in relation to *Leishmania* infection in dogs [20, 21, 23, 24] and mice [25] because of its immunomodulatory effects. The origin of the effects of domperidone is related to the release of serotonin, which causes a reversible increase in blood levels of prolactin [26]. Prolactin has been classified as a pro-inflammatory lymphocyte-derived cytokine [27], and its increase induces a boost of CD4^+^ T lymphocytes, in addition to the release of cytokines such as IL-2, IFN-γ and TNF-α, producing activation of natural killer (NK) and macrophages, followed by a decrease in CD4^+^ Th2 and TNF-β [28–30].

The use of domperidone has been studied in healthy [24, 31] and sick dogs with leishmaniosis [20, 21, 23]. A lower risk of developing clinical leishmaniosis in healthy seronegative dogs was observed compared to dogs left untreated [24]. In dogs with clinical leishmaniosis, a reduction of clinical signs was observed in those that presented a mild disease [20] while a reduction of serum creatinine, globulins, gamma globulins, anti-*L. infantum* antibody titers and C-reactive protein was observed in dogs with leishmaniosis affected by chronic kidney disease [21]. Moreover, dogs with clinical leishmaniosis that were treated with a combination of furazolidone and domperidone showed a reduction of skin lesions [23]. However, no studies have yet been published in healthy *L. infantum*-seropositive dogs treated with domperidone.

As stated previously, a broad range of immune responses and clinical manifestations have been described in canine *L. infantum* infection, and an important number of dogs are seropositive and healthy [5]. For example, the seroprevalence of *L. infantum* in Spain has been reported to be near 10% [32–34], although the prevalence of dogs that develop the clinical disease is usually < 10% in the infected dogs [2, 35]. These healthy *L. infantum*-seropositive dogs pose a public health issue as they can maintain the domestic cycle of *Leishmania* and thus increase the risk of infection to other dogs [2, 5]. In addition, these healthy *L. infantum*-seropositive dogs have a high risk of developing clinical leishmaniosis in the future [36, 37]. Moreover, healthy *L. infantum*-seropositive dogs are usually scientifically neglected, and few recommendations have been published such as using repellents all year round, monitoring without treatment, short treatments with conventional anti-*Leishmania* drugs or immunotherapy [2, 5, 38]. Nonetheless, there is still limited evidence for treatment outcomes for these dogs, and the efficacy of these recommendations remains inconclusive [5].

Therefore, there is still limited information regarding the use, efficacy and safety of immunotherapy using domperidone in the clinical setting. The aim of this study was to evaluate the clinical efficacy and safety of domperidone (Leisguard^®^) as immunotherapy in *Leishmania*-seropositive healthy dogs.

## Methods

### Clinical trial design

This was a blinded, randomized and controlled multicenter clinical trial with a 1-year follow-up. The study started on September 2020, and the last sample was received in July 2022. The enrollment period was from September 2020 to June 2021.

The clinical trial was performed in a total of 48 centers divided into 38 veterinary practices and 10 dog shelters from various regions of the Iberian Peninsula, a region with reported average seroprevalences of *L. infantum* infection near 10% [32–34, 39].

### Treatments and randomized assignment

The clinical trial included two groups with different treatments. One group was treated with domperidone (Leisguard^®^) and named treated group (TG) while the other group was treated with a placebo and named control group (CG). Both domperidone and placebo were administered orally. The composition of placebo included the same excipients of domperidone (Leisguard^®^): methyl parahydroxybenzoates (E218 and E216) and quinolone yellow (E104). The dose of domperidone was 0.5 mg/kg, or its equivalent, for placebo in volume, once daily, during 4 consecutive weeks. Treatment was administered by the owner or caregiver of the dog and repeated every 4 months until the end of the 1-year follow-up period. Both domperidone and placebo had to be administered mixed with food or administered directly in the mouth of the dog.

Domperidone and placebo had the same appearance and were labeled as treatment A or treatment B. Therefore, treatment administration was blinded. Dog owners or caregivers, veterinarians and laboratory personnel running tests were blinded. The study director was in charge of the randomization lists and thus was the only non-blinded researcher in the clinical trial. Veterinarians were instructed about which product (A or B) they were going to administer to each dog, but neither the veterinarians nor the owners of the dog(s) had knowledge about which product was being administered. Furthermore, the owners or caregivers of the dog(s) had to fill in a data collection form to record both the daily treatment administration and any adverse drug reaction or lack of efficacy occurring during the study. Laboratory personnel received samples with dog data, but no information about the treatment the dog received.

A randomized assignment of the treatment was also performed. Block randomization was performed creating a randomly generated list of 15 treatments with a 2:1 ratio for all centers, so for each two dogs included in the TG, only one dog was included in the CG. Leisguard^®^ and placebo were then distributed in all sites of the clinical trial following the previously generated list. When the site distributed all the received treatments, a new randomly generated list was prepared, and new treatments were distributed.

### Sample size

To estimate differences between percentages of the two treatment groups (TG and CG), the necessary sample size was calculated based on the proportions of the parameter of interest in the groups, confidence level and power [40]. The sample size was calculated for unilateral tests, with a 2:1 proportion, confidence level of 95%, 80% of power and potential dropout of 20% [40]. The sample size required was 116 dogs in the TG and 58 dogs in the CG. Hence, the total sample size required was 174 dogs.

### Dog selection and inclusion/exclusion criteria

Dogs of different sexes (entire or neutered), breeds (pure breed or crossbreed), ages, weights and living situation (client-owned dogs or dogs from shelters) were able to be enrolled in the clinical trial. Female dogs known to be pregnant or lactating were not able to be enrolled.

The inclusion criteria included the following characteristics: (i) not previously diagnosed with clinical leishmaniosis, (ii) presenting a recent seropositive result for the detection of *L. infantum* antibodies and (iii) being healthy (not showing clinical signs or clinicopathological abnormalities compatible with leishmaniosis). Dogs were considered healthy when they did not present clinical signs or clinicopathological abnormalities based on a physical examination and complete blood count (CBC), biochemistry profile and urinalysis including urinary protein creatinine ratio (UPC). CBC, biochemistry and urinalysis had to be within reference intervals. However, a slight variation outside the reference intervals (always no more than 5%) was evaluated individually. Then, whether the results were truly of clinical relevance or not to the patient was assessed.

The exclusion criteria included the following characteristics: (i) poor body condition such as dogs with very low body condition score (evident bony prominences, no palpable fat, amyotrophy), (ii) had been previously treated with anti-*Leishmania* drugs (meglumine antimoniate, allopurinol, miltefosine, etc.), immunomodulators (Leisguard^®^, Impromune^®^…) or vaccines against CanL (CaniLeish^®^, Letifend^®^), (iii) were recently treated (at least the last month) with drugs that could affect the outcome of the disease or the action of domperidone such as immunosuppressive drugs (corticosteroids, azathioprine, cyclosporine, tacrolimus), antibiotics (quinolones) and dopaminergic drugs (dopamine, dobutamine, cabergoline) and (iv) incapacity to follow a 1-year treatment or to comply with the follow-up visits or treatment administration.

### Withdrawal criteria

Dogs had to be withdrawn from the study when at least one of the following situations occurred: (i) presence of an adverse drug reaction that compromised the health of the treated dog and the ongoing treatment, (ii) appearance of clinical signs or clinicopathological abnormalities of leishmaniosis, specifically when needing anti-*Leishmania* treatment, (iii) need of other treatments that could interfere with the results of the clinical trial, when interfering in the outcome of the infection or the action of domperidone (quinolones, cabergoline, omeprazole, cimetidine, dopamine, dobutamine, corticosteroids, etc.) and (iv) pregnant or lactating females.

### Study flowchart

**Fig. 1:**
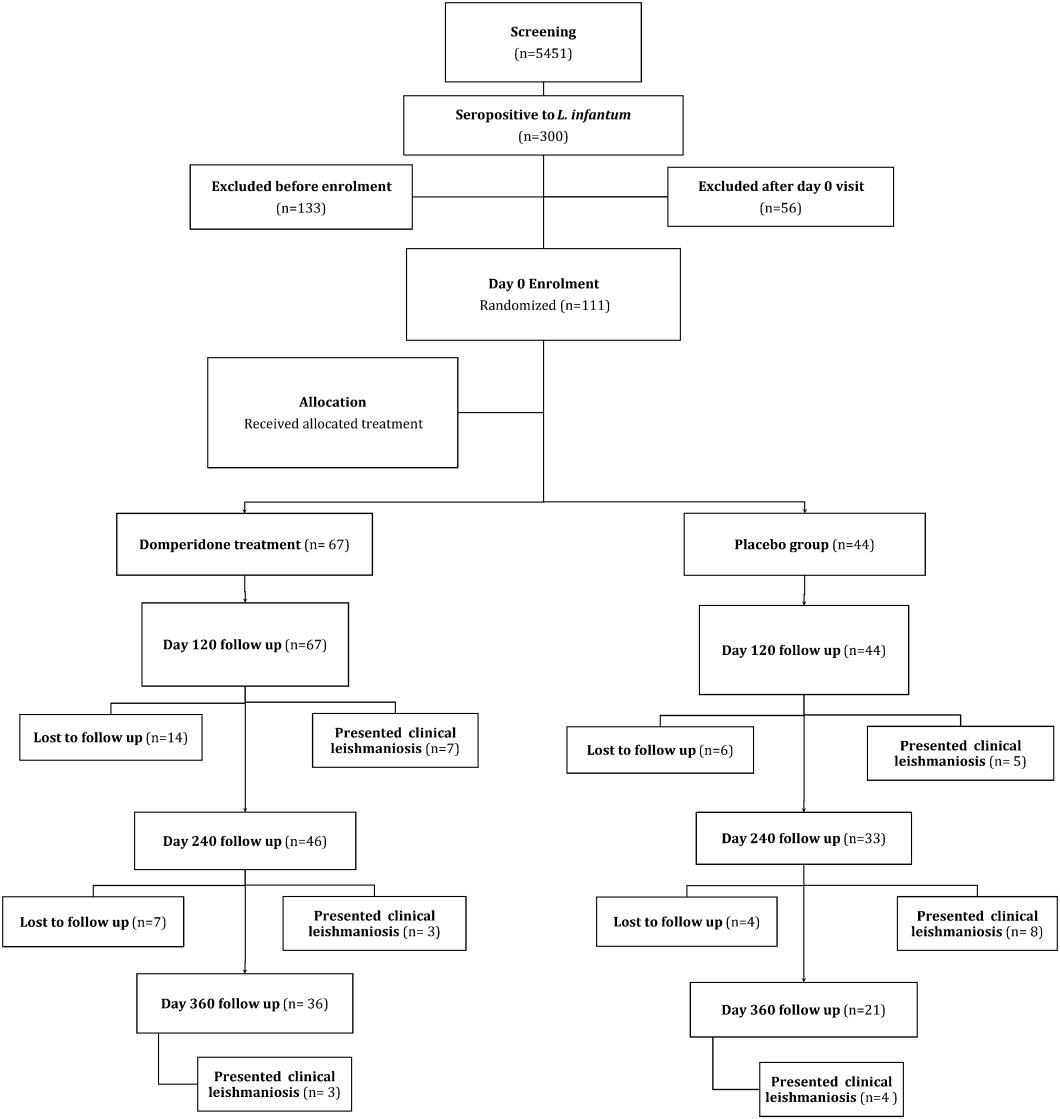
Flowchart displaying the number of dogs screened, recruited, lost to follow-up and analyzed

A flowchart of the study is depicted in Fig. 1. An initial screening was performed to diagnose healthy *L. infantum* seropositive dogs. This initial screening included a procedural protocol with indications on how to perform the physical examination to confirm that the dog was healthy and how to collect blood samples. All veterinarians received data collection forms for the registration of all procedures. The blood sample was used to test for anti-*Leishmania* antibodies; first a commercial ELISA was performed (Leiscan^®^, Ecuphar veterinaria SLU, Spain), and all doubtful and seropositive samples were further investigated by a quantitative in-house ELISA [41]. A screening of 5451 apparently healthy dogs was performed, and 300 dogs (5.5%) were seropositive to *L. infantum* (Fig. 1). After this screening, blood and urine samples were taken again for CBC, biochemistry profile and urinalysis with UPC to better assess clinical status of *L. infantum*-seropositive dogs prior to enrollment. Healthy seropositive dogs with no clinicopathological abnormalities were included in the study (*n* = 111). Exclusion before enrollment of these dogs (*n* = 133) was mainly due to presence of clinicopathological abnormalities (*n* = 116) or incapacity to follow a 1-year treatment or to comply with the follow-up visits (*n* = 17). Exclusion after day 0 visit was mainly due to incapacity to follow a 1-year treatment or to comply with the follow-up visits (*n* = 56).

Most dogs included in the study (*n* = 70) were enrolled during non-sandfly transmission season (December to March) [32] while the others (*n* = 41) were enrolled during sandfly season (April to November) [32].

### Examination and sampling

The physical examination and data collection were performed on days 0, 30, 120, 150, 240, 270 and 360 by veterinary general practitioners.

An initial clinical evaluation of the dog was performed on day 0 by the veterinarian to confirm that the dog was healthy and could be enrolled in the clinical trial. First, the clinical history of the dog was recorded with information about the signalment (breed, age, sex, reproductive status, lifestyle: housed or kennelled, diet) and clinical history (vaccination status, previous or current diseases, current medications). The information registered was general appearance (mental status, attitude, body condition, hydration, body weight, temperature and heart/pulse rate) and description of any abnormalities or lesions noted and presence of external parasites on physical examination. The clinical evaluation was then repeated on days 30, 120, 150, 240, 270 and 360. A clinical evaluation was also performed in cases of early withdrawal and for any dog experiencing a serious adverse drug reaction. Dogs that showed any adverse drug reaction due to treatment or evidence of illness were closely monitored as needed throughout the study.

Samples of blood and urine were collected on days 0, 120, 240 and 360 for further laboratorial tests. Blood samples were collected by jugular, cephalic or metatarsian venepuncture. Urine was obtained by free catch or cystocentesis. Once collected, all samples were refrigerated until shipment. Shipment was performed no later than 24–48 h after sample collection.

On days 30, 150 and 270, the veterinarian confirmed treatment compliance and that the dog was still healthy after the administration of the treatment. The information was obtained in a consultation or by phone call.

Furthermore, a spot-on repellent as a combination of permethrin and pyriproxyfen (Advantix®, Elanco Animal Health, USA) was applied in all dogs during the 1-year follow-up, every 3 weeks.

### Routine laboratory tests

Routine laboratory tests were performed at days 0, 120, 240 and 360 at the referral private veterinary diagnostic laboratory (Laboratorio Echevarne: https://laboratorioechevarne.com/en/veterinary/). The investigated parameters are specified in Table 1. The hematology panel was performed with XN1000 SYSMEX (Sysmex España SL, Spain), biochemistry panel and urinary protein creatinine ratio (UPC) were performed with VITROS 5600 ORTHO (Ortho Clinical Diagnostics, USA), serum electrophoresis was performed with CAPILLARYS 3 SEBIA (Sebia, Hispania SA, Spain), and urine panel (except UPC) was performed with BECKMAN strips (Beckman Coulter, USA). Urine density, physical characteristics and sediment analysis were also investigated. Reference intervals of each parameter are also depicted in Table 1.

**Table 1:** Parameters of routine blood and urine tests and reference intervals

Hematology panel	Reference intervals [42, 43]	Biochemistry panel	Reference intervals [44, 45]
RBC (10^12^/l)	5.1–7.6	Total protein (g/l)	54–71
Hemoglobin (g/dl)	12.4–19.2	Albumin (g/l)	26–33
Hematocrit (%)	35–52	Globulin (g/l)	27–44
MCV (fl)	60–77	A/G ratio	0.86–1.93
MCH (pg)	21.9–26.3	ALT (U/l)	21–102
MCHC (g/dl)	34.4–38.1	ALP (U/l)	20–156
WBC (10^9^/l)	5.6–20.4	Creatinine (mg/dl)	0.5–1.5
Neutrophils conc (10^9^/l)	2.9–13.6	Urea (mg/dl)	21.4–59.9
Lymphocyte concentration (10^9^/l)	1.1–5.3	Serum electrophoresis (g/l)	Sero-albumin (24.4–49.6); alpha-1 globulin (1.7–4.5); alpha-2 globulin (3.8–10.2); beta globulin (8–18); gamma globulin (2.6–11.7)
Monocytes conc (10^9^/l)	0.4–1.6	Urinary panel	Reference intervals [46]
Eosinophils conc (10^9^/l)	0.1–3.1	Urine strip*	
Basophils conc (10^9^/l)	0–0.2	UPC	< 0.5
Platelet conc (10^9^/l)	200–500	Density (g/l)	1006-1050
Evaluation of blood smear		Physical color and appearance, microscopic appearance and sediment analysis	

*A/G* albumin/globulin; *ALP* alkaline phosphatase; *ALT* alanine transaminase; *MCH* mean corpuscular hemoglobin; *MCHC* mean corpuscular hemoglobin concentration; *MCV* mean corpuscular volume; *RBC* red blood cell concentration; *UPC* urinary protein creatinine ratio; *WBC* leukocyte concentration

^*^Urine strip included qualitative information about density, acetone, pH, proteins, blood, nitrites, glucose, urobilinogen, urobilin and leukocytes

### Quantitative in-house ELISA for the detection of *L. infantum*-specific antibodies

An in-house ELISA was performed on sera at days 0, 120, 240 and 360 as previously described [41]. Samples were first diluted to 1:800 in phosphate-buffered saline (PBS)-Tween with 1% dry milk and then incubated 1 h at 37 °C. After the washes, peroxidase conjugated Protein A (Peroxidase Conjugate Protein A; Merck KGaA, Germany) at a concentration of 0.16 ng/µl was added to the plate and incubated 1 h at 37 °C. After washes, *o*-phenylenediamine and substrate buffer (SIGMAFAST OPD; Merck KGaA, Germany) were added to the plate. The reaction was stopped with 5 M H2SO4. Plates were read at 492 nm in a spectrophotometer machine (MB-580 HEALES; Shenzhen Huisong Technology Development Co., Ltd, China) and were defined as ELISA units (EU) in relation with a positive canine sera sample used as a calibrator set at 100 EU. The cutoff of the sera in-house ELISA was already determined at 35 EU using the ELISA results of 80 dogs from a non-endemic area as previously described [47]. Furthermore, sera were classified as high positive when having a result ≥ 300 EU, medium positive when having a result ≥ 150 EU and < 300 EU, low positive when having a result ≥ 35 EU and < 150 EU, and negative when having a result < 35 EU.

All samples classified as medium or high positive were further studied using a two-fold serial dilution ELISA. Sera two-fold dilutions were started at 1:800 and continued for 7 to 11 further dilutions. The result was also quantified as EU related to a calibrator arbitrary set at 100 EU, with an optical density (OD) value of one at the 1:800 dilution. The mean values of the dilutions at which the OD were close to one were chosen for the calculation of the EU using the following formula: (Sample OD/Calibrator OD) × 100 × dilution factor.

### Blood DNA extraction and *Leishmania* real-time PCR

Blood DNA extraction was performed with a commercial blood DNA extraction kit (MagMAX CORE Nucleic Acid Purification Kit, Thermo Fisher Scientific Inc., USA) using an automated system (KingFisher Flex Purification System, Thermo Fisher Scientific Inc., USA) following the manufacturer’s instructions for a simple workflow with whole EDTA-blood samples. *Leishmania* real-time (RT-) PCRs were performed as described elsewhere [36, 48–50] at the beginning and end of the study. Amplifications were performed in triplicate for each sample. Positive and negative controls were also included in each plate. A tenfold dilution series of standard DNA from promastigotes (MHOM/ES/2016/CATB101; *L. infantum* ZMON-1) was used as a calibrator (serial dilution from 10^5^ parasites/ml to 10^–3^ parasites/ml), allowing for the plotting of a standard curve [36]. Results were considered positive when the quantification cycle (Cq) was < 40 and the amplification was detected in all the replicates [36]. A total of 84 dogs of the study were analyzed (51 of the TG and 33 of the CG).

### Whole-blood stimulation assay (WBA) and IFN-γ concentration

Whole-blood stimulation assay (WBA) and IFN-γ concentration were performed at the beginning and end of the study as described elsewhere [51].

Briefly, 300 μl heparinized whole blood was analyzed separately in three conditions: medium alone, medium with soluble *L. infantum* antigen (*L. infantum* antigen 1 mg/ml) at a concentration of 10 μg/ml and medium with mitogen concanavalin A (100 mg Medicago®, Sweden) at a concentration of 10 μg/ml. Incubation lasted for 5 days at 37 °C in 5% CO_2_ environment. After incubation, blood was collected in sterile tubes and centrifuged at 300 g for 10 min, and supernatants were collected and stored at – 80ºC until further use.

IFN-γ was determined in the collected supernatants by a commercial sandwich ELISA following the manufacturer’s instructions (DuoSet® ELISA, R&D Systems, USA). Standard curve for IFN-γ started at 8000 pg/ml, and two-fold dilutions were made until 62.5 mg/ml. Supernatants containing concanavalin A stimulation were diluted at a proportion 1:1 with reagent diluent provided by the manufacturer. The results were read at 450 nm in a spectrophotometer machine (MB-580 HEALES; Shenzhen Huisong Technology Development Co., Ltd., China) and processed using a four-parameter logistic curve provided by MyAssays program (http://www.myassays.com/). Plates were repeated when the R^2^-value of the standard curve was < 0.98. Samples from each dog (at the beginning and at the end of the study) were analyzed on the same plate in duplicates. A total of 82 study dogs were analyzed (47 of the TG and 35 of the CG).

### Efficacy variables

Efficacy variables were classified in two groups: primary and secondary outcomes. The primary outcome focused on the development of the disease; thus, dogs were classified as healthy or sick. Dogs were considered healthy when they did not present clinical signs or clinicopathological abnormalities based on physical examination and hematology, biochemistry profile and urinalysis. Furthermore, all sick dogs were classified using the LeishVet clinical stage for clinical leishmaniosis [10]. Antibody detection against *Ehrlichia canis*, *E. ewingii*, *Anaplasma phagocytophilum*, *A. platys*, *Borrelia burgdorferi* antigens and *Dirofilaria immitis* antigen (SNAP 4Dx Plus®, IDEXX, USA) were investigated in all dogs that developed disease. Moreover, sick dogs were treated for clinical leishmaniosis with a conventional anti-*Leishmania* treatment [5, 52] chosen at discretion of the veterinarian in charge of the dog.

The secondary outcome focused on quantitative in-house ELISA and its changes between days (0, 120, 240 and 360) and *Leishmania* real-time PCR and IFN-γ concentrations at day 0 and at the end of the study. Thus, a change was reported when the results between days presented a significant increase or decrease. Furthermore, seroreversion (changing from a seropositive result to a seronegative) in endpoint in-house ELISA for *L. infantum* was also investigated between days (0, 120, 240 and 360).

### Safety evaluation

Adverse drug reaction evaluation was used to demonstrate the safety of the products. An adverse drug reaction was defined as any observation in the treated dog that was unfavorable, unintended and occurred after the administration of the product. The adverse drug reaction was immediately registered with a detailed description and, depending on the severity of the adverse drug reaction, the treatment could be interrupted. Dogs that showed adverse drug reactions were closely monitored as needed throughout the clinical trial and withdrawn if necessary. This information was recorded in the data collection form of both the veterinarian and the owner or caregiver of the dog.

### Statistical analysis

The statistical analysis was performed using the package Stats for the software R i386 3.6.1 for Windows, using Fisher’s exact test for qualitative variables and Mann-Whitney *U* test for quantitative variables to compare between treatment groups (TG vs. CG) and seropositivity groups (low seropositive vs. medium to high seropositive). Analysis was performed following the intention-to-treat principle [53]. Log-rank test (a survival analysis) was performed to detect differences between the event curves of treatment groups (TG vs. CG) with the studied event being the occurrence of disease development. Wilcoxon signed-rank test was performed to compare the results of quantitative in-house ELISA, *Leishmania* real-time PCR and IFN-γ concentration between the beginning of the study (day 0) and other time points (day 120, 240 and 360). The Shapiro-Wilk test was performed to detect normal distribution of quantitative variables. A *P*-value < 0.05 was considered statistically significant. Graphs were plotted using Graphad Prism version 5.00 for Windows (GraphPad Software, San Diego, CA, USA).

## Results

### Signalment and clinical data

All raw data of the clinical trial can be found as additional file 1. Characteristics of all dogs included in the clinical trial are depicted in Table 2. The most common breeds in the TG were Labrador retriever (7.5%) and Spanish greyhound (4.5%) while German shepherd (6.8%), Beagle (4.5%) and Jagdterrier (4.5%) were the most common in the CG. No differences were found between treatment groups between breed, sex, lifestyle, age and weight (Table 2).

**Table 2:** Baseline characteristics of the dogs assigned to each study group

Qualitative characteristics		Total (*n* = 111) % (95% CI)	TG (*n* = 67) % (95% CI)	CG (*n* = 44) % (95% CI)	*P*-value (Fisher’s exact test)
Breed	Crossbreed	47.7 (38.2–57.4)	53.7 (41.1–66)	38.6 (24.4–54.5)	0.127
	Purebred	52.3 (42.6–61.8)	46.3 (34–58.9)	61.4 (45.5–75.6)	
Sex	Female	44.1 (34.7–53.8)	47.8 (35.4–60.3)	38.6 (24.4–54.5)	0.435
	Male	55.9 (46.1–65.3)	52.2 (39.7–64.6)	61.4 (45.5–75.6)	
Lifestyle	Housed	78.4 (69.6–85.6)	76.1 (64.1–85.7)	81.8 (67.3–91.8)	0.638
	Kennelled	21.6 (14.4–30.4)	23.8 (14.3–35.9)	18.2 (8.2–32.7)	
ELISA interpretation at day 0	High or medium positive	38.7 (29.6–48.5)	34.3 (23.2–46.9)	45.5 (30.4–61.2)	0.319
	Low positive	61.3 (51.6–70.4)	65.7 (53.1–76.9)	54.6 (38.9–69.6)	
*Leishmania* real-time PCR	Positive	35.7 (25.6–46.9)	33.3 (20.8–47.9)	39.4 (22.9–57.9)	0.644
	Negative	64.3 (53.1–74.5)	66.7 (52.1–79.2)	60.6 (42.1–77.1)	

Quantitative characteristics		Median (min–max)	Median (min–max)	Median (min–max)	*P*-value (Mann-Whitney *U* test)
Age (years)		5 (1–14)	5 (1–14)	4 (1–13)	0.094
Weight (kg)		24 (6–55)	22 (6–50)	25 (10–55)	0.193
Endpoint ELISA (EU) at day 0		165 (40–3965)	155 (40–1954)	183 (55–3965)	0.320
IFN-γ concentration (pg/ml)	Medium with soluble *Leishmania infantum* antigen	447 (0–14190)	252 (0–14190)	834 (0–9418)	0.452
	Medium with concanavalin A	6833 (860–43290)	7051 (2057–43290)	5629 (860–23900)	0.062

*CG* control group; *CI* confidence interval, *EU* ELISA units; *max* maximum; *min* minimum; *TG* treated group

In-house ELISA results and interpretation of all dogs at initial day 0, and also classified by treatment group (TG and CG), are depicted in Table 2. There was a high percentage of low-seropositive dogs included in the study (61.3%) while 38.7% were medium or high seropositive (Table 2). No differences were detected between treatment groups when comparing in-house ELISA results and their interpretation at day 0 (Table 2).

A total of 31 dogs (28%, 31/111) were lost to follow-up during the study (Fig. 1). Specifically, the dropout rate in the TG was 31% (21/67), while it was 23% (10/44) in the CG. No significant differences in dropout rates were observed between groups (*P* > 0.05). Of these dogs, 20 (14 in the TG and 6 in the CG) were lost to follow-up after day 120, and 11 dogs (7 in the TG and 4 in the CG) were lost after day 240. These dogs were lost to follow-up mainly because of adoption (*n* = 13), owner decision (*n* = 10), moving to another region (*n* = 2) and other causes such as sudden death by a car accident (*n* = 2), detection of other diseases (meningioma and carcinoma) (*n* = 2) or pregnancy (*n* = 1).

### Efficacy variables

#### Primary outcome

Thirty dogs developed disease during the follow-up period. Information about signalment, immunological and parasitological status, clinical findings and LeishVet clinical staging is summarized in Additional file 2: Table S1. Thirteen (13/67; 19.4%) were from the TG while the other 17/44 (38.6%) were from the CG. A significant difference was observed (Fisher’s exact test: *P* = 0.03, OR = 2.62, CI 1.11–6.17), which indicated that the TG was less likely to present disease development compared to the CG. Most of the dogs that developed disease presented clinicopathological abnormalities while a minority also presented clinical signs. The specific clinical signs and clinicopathological abnormalities that the dogs developed are depicted in Table 3. SNAP 4Dx Plus® (IDEXX, USA) tests were negative in all dogs that developed disease. Dogs were classified by LeishVet clinical staging: 22 dogs (9 of the TG and 13 of the CG) were in stage IIa, 2 dogs of the TG were in stage IIb, 1 dog of the TG was in stage III and 2 dogs (1 of the TG and 1 of the CG) were in stage IV (Additional file 2: Table S1) [10]. Three dogs of the CG were not classified by LeishVet clinical staging because of lack of urinalysis. At the beginning of the study, the majority of dogs that developed disease were classified in the medium- to high-seropositive group (63.3%) with a median of the endpoint ELISA of 277 EU with a minimum of 57 EU and a maximum of 1774 EU. The median of the endpoint ELISA performed at day of failure in the dogs that developed disease was 1629 EU with a minimum of 36 EU and a maximum of 6151 EU.

**Table 3:** Clinical signs and clinicopathological abnormalities of dogs that developed disease

Clinical signs	Total (*n* = 30) number of cases (%)	TG (*n* = 13) number of cases (%)	CG (*n* = 17) number of cases (%)
Skin lesions (scales, ulcers and alopecia)	8 (26.7)	4 (30.8)	4 (23.5)
Weight loss	5 (16.7)	4 (30.8)	1 (5.9)
Generalized lymphadenomegaly	4 (13.3)	2 (15.4)	2 (11.8)
Conjunctivitis	1 (3.3)	0 (0)	1 (5.9)
Total	9 (30)	4 (30.8)	5 (29.4)

Clinicopathological abnormalities	Total (*n* = 30) number of cases (%)	TG (*n* = 13) number of cases (%)	CG (*n* = 17) number of cases (%)
Hyperproteinemia	27 (90)	12 (92.3)	15 (88.2)
Hyperglobulinemia - hypergammaglobulinemia - hyperbetaglobulinemia - hyperalfaglobulinemia	28 (93.3) 26 (86.7) 11 (36.7) 6 (20)	13 (100) 12 (92.3) 7 (53.9) 3 (23.1)	15 (88.2) 14 (82.4) 4 (23.5) 3 (17.6)
Decreased A/G ratio	22 (73.3)	11 (84.6)	11 (64.7)
Mild normocytic normochromic anemia non-regenerative	6 (20)	3 (23.1)	3 (17.6)
Proteinuria	5 (16.7)	4 (30.8)	1 (5.9)
Prerenal or renal azotemia	2 (6.7)	1 (7.7)	1 (5.9)
Leukocytosis with mature neutrophilia	1 (3.3)	0 (0)	1 (5.9)
Total	29 (96.7)	13 (100)	16 (94.1)

*A/G* albumin/globulin; *CG* control group; *TG* treated group

When the dogs were classified not only by their treatment group, but also by their initial in-house ELISA result (low-positive vs. medium- to high-seropositive), different outcomes were observed. In the low-seropositive group (*n* = 68), a total of 11 dogs developed disease, 4 in the TG (4/44, 9.1%) and 7 in the CG (7/24, 29.2%). Thus, low-seropositive dogs treated with domperidone were significantly less likely to develop disease compared to low-seropositive dogs treated with placebo (Fisher’s exact test: *P* = 0.04, OR = 4.12, CI 1.06-15.94). In the medium- to high-seropositive group (*n* = 43), a total of 19 dogs developed disease 9 in the TG (9/23, 39.1%) and 10 in the CG (10/20, 50%). Thus, no differences were found between treatments in medium- to high-seropositive dogs (*P* > 0.05).

**Fig. 2:**
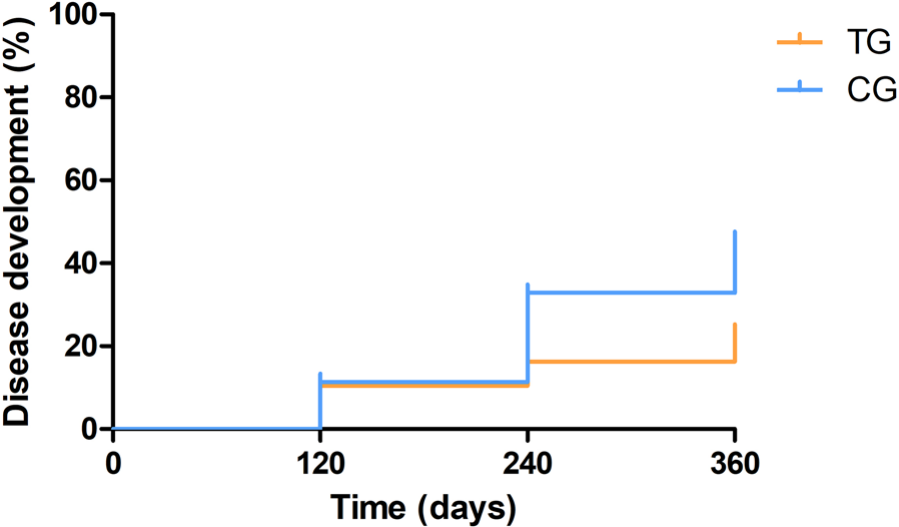
Disease development curves by treated groups of all dogs (log-rank test: *X*^2^ = 4.03, *df* = 1, *P* = 0.04). *CG* control group; *TG* treated group

**Fig. 3:**
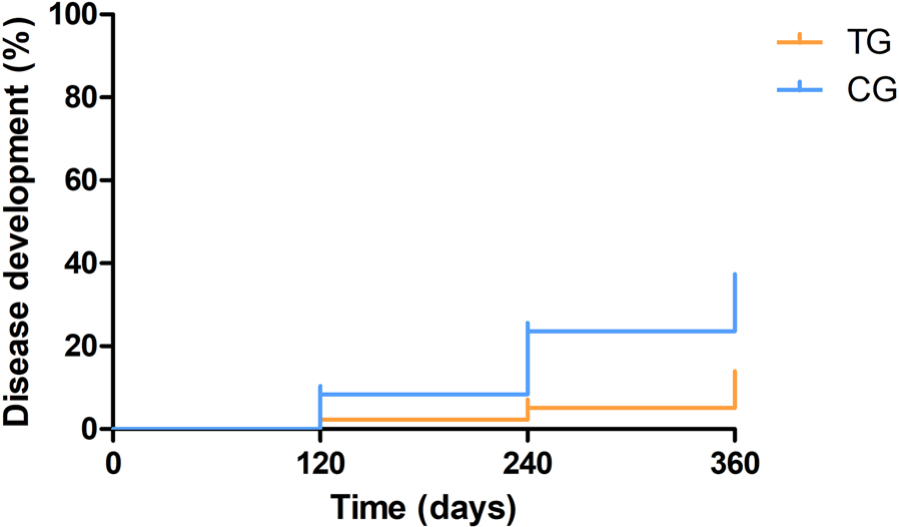
Disease development curves by treated groups of low-seropositive dogs (log-rank test: *X*^2^ = 4.67, *df* = 1, *P* = 0.03). *CG* control group; *TG* treated group

**Fig. 4:**
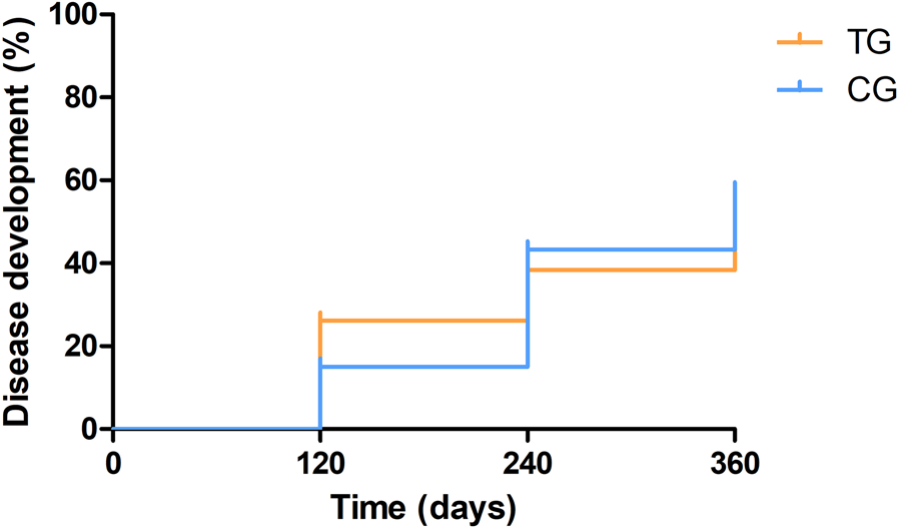
Disease development curves by treated groups of medium- to high-seropositive dogs (log-rank test: *X*^2^ = 0.05, *df* = 1, *P* = 0.83). *CG* control group; *TG* treated group

When a log-rank test was performed (Fig. 2), a significant difference between the disease development curves was observed between the TG and the CG (log-rank test: *X*^2^ = 4.03, *df* = 1, *P* = 0.04). The disease development curve in the TG group presented a proportion of 23.4% at day 360 while the CG presented a proportion of 45.6% at day 360 (Fig. 2). When the dogs were classified not only by their treatment group but also by their initial in-house ELISA result (low positive vs. medium to high seropositive), different outcomes were also observed. In the low-seropositive group, a significant difference between the disease development curves was observed between the TG and the CG (log-rank test: *X*^2^ = 4.67, *df* = 1, *P* = 0.03). In this case, the TG curve presented a proportion of 11.9% at day 360 while the CG curve presented 35.4% at day 360 (Fig. 3). In the medium- to high-seropositive group, no difference between the disease development curves was observed between the TG and the CG (*P* > 0.05). In this case, the TG curve presented a proportion of 46.1% at day 360 and the CG curve presented a 57.5% at day 360 (Fig. 4).

#### Secondary outcome

**Fig. 5:**
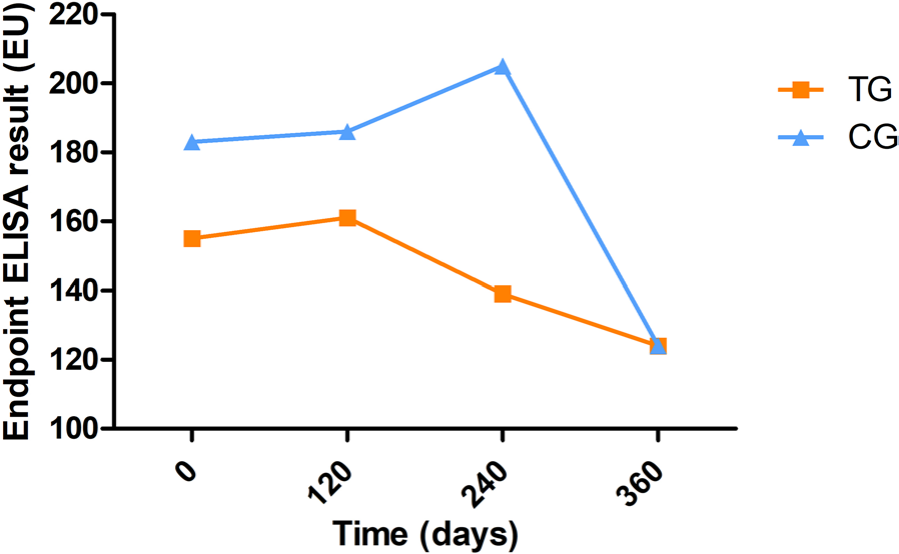
Median endpoint ELISA results of the dogs by treated groups at days 0, 120, 240 and 360. *CG* control group; *EU* ELISA units; *TG* treated group

The medians of in-house ELISA results of each day (0, 120, 240 and 360) classified by treatment group are given in Fig. 5. No differences in in-house ELISA results were detected between the studied days and treatment groups (*P* > 0.05) (Additional file 3: Table S2). Seroreversion was observed in 10 dogs (10/111; 9%) during the study: 8 (8/67; 11.9%) from the TG and 2 (2/44; 4.5%) from the CG. All these dogs were classified as low positive at day 0. Only three of these dogs (3/111; 2.7%) maintained seroreversion until the end of the study: two (2/67; 3%) from the TG and one (1/44; 2.3%) from the CG. No differences were observed between groups (*P* > 0.05). Further information about the number of seronegative dogs at each visit and whether they were sampled during sandfly season is available in Additional file 4: Table S3.

The in-house ELISA results of the dogs with disease development of each day (0, 120, 240 and 360) by treatment group are depicted in Table 4. The in-house ELISA results were significantly higher at days 120 and 240 compared to day 0 (Table 4). In the TG, the in-house ELISA results were also significantly higher at days 120 and 240 compared to day 0 while, in the CG, the in-house ELISA results were only significantly higher at day 120 compared to day 0 (Table 4).

**Table 4:** Endpoint ELISA results of the dogs with disease development at day 0, 120, 240 and 360

Group (number of dogs)	Endpoint ELISA result (EU) median (min–max)			
	Day 0	Day 120	Day 240	Day 360
Total (*n* = 30)	277 (57–1774)	819 (23–5287)^a^	816 (19–4415)^b^	373 (36–6151)
TG (*n* = 13)	295 (107–1774)	915 (197–5287)^c^	1556 (195–2910)^d^	2939 (457–6151)
CG (*n* = 17)	258 (57–1720)	545 (23–4344)^e^	550 (19–4415)	150 (36–373)

*CG* control group; *EU* ELISA units; *max* maximum; *min* minimum; *TG* treated group

^a^Significantly higher compared to day 0 (Wilcoxon signed-rank test: W: − 423, *P* < 0.0001)

^b^Significantly higher compared to day 0 (Wilcoxon signed-rank test: W: − 133, *P* = 0.004)

^c^Significantly higher compared to day 0 (Wilcoxon signed-rank test: W: − 91, *P* = 0.0002)

^d^Significantly higher compared to day 0 (Wilcoxon signed-rank test: W: − 21, *P* = 0.031)

^e^Significantly higher compared to day 0 (Wilcoxon signed-rank test: W: − 121, *P* = 0.005)

The *Leishmania* real-time PCR results at the beginning and end of the study of all dogs and by treatment groups are depicted in Table 5. A significantly higher proportion of PCR positive dogs in the CG group were observed at the end of the study compared to the TG group, but no differences were observed between median parasites/ml results (Table 5). When dogs were classified by their initial in-house ELISA result (low positive vs. medium to high seropositive), a significantly higher proportion of PCR-positive dogs in the medium- to high-seropositive group were observed at the end of the study compared to the low-seropositive group (Table 6). Furthermore, Ct results of medium- to high-seropositive dogs were significantly different at the beginning and end of the study presenting a higher median parasite burden (Table 6).

**Table 5:** *Leishmania* PCR results of the dogs at the beginning and end of the study

Group (number of dogs)	PCR results at the beginning		PCR results at the end	
	Positive dogs (%)	Median parasites/ml (min–max)	Positive dogs (%)	Median parasites/ml (min–max)
Total (*n* = 84)	30 (35.7)	0.02 (0.004–5.86)	29 (34.5)	0.5 (0.004–502.4)
TG (*n* = 51)	17 (33.3)	0.01 (0.004–3.29)	13 (25.5)^a^	1.69 (0.013–502.4)
CG (*n* = 33)	13 (39.4)	0.03 (0.004–5.86)	16 (48.5)^a^	0.28 (0.004–83)

*CG* control group; *Ct* cycle threshold; *max* maximum; *min* minimum; *TG* treated group

^a^Proportion of PCR-positive dogs in the CG group was significantly higher compared to the TG (Fisher’s exact test: *P* = 0.037, OR = 2.75, CI 1.09–7)

**Table 6:** *Leishmania* PCR results of seropositive dogs at the beginning and end of the study

Group (number of dogs)	PCR results at the beginning		PCR results at the end	
	Positive dogs (%)	Median parasites/ml (min–max)	Positive dogs (%)	Median parasites/ml (min–max)
Low seropositive (*n* = 54)	17 (31.5)	0.01 (0.004–5.86)	12 (22.2)^a^	0.02 (0.004–83)
Medium to high seropositive (*n* = 30)	13 (43.3)	0.03 (0.005–3.29)^b^	17 (56.7)^a^	2.12 (0.005–502.4)^b^

*Ct* cycle threshold; *max* maximum; *min* minimum

^a^Proportion of PCR-positive dogs in the medium- to high-seropositive group was significantly higher compared to the low-seropositive group (Fisher’s exact test: *P* = 0.002, OR = 4.58, CI 1.74–12.03)

^b^Medium- to high-seropositive group presented a significant difference of median PCR at the beginning and end of the study (Wilcoxon signed-rank test: W: 30, *P* = 0.039)

**Fig. 6:**
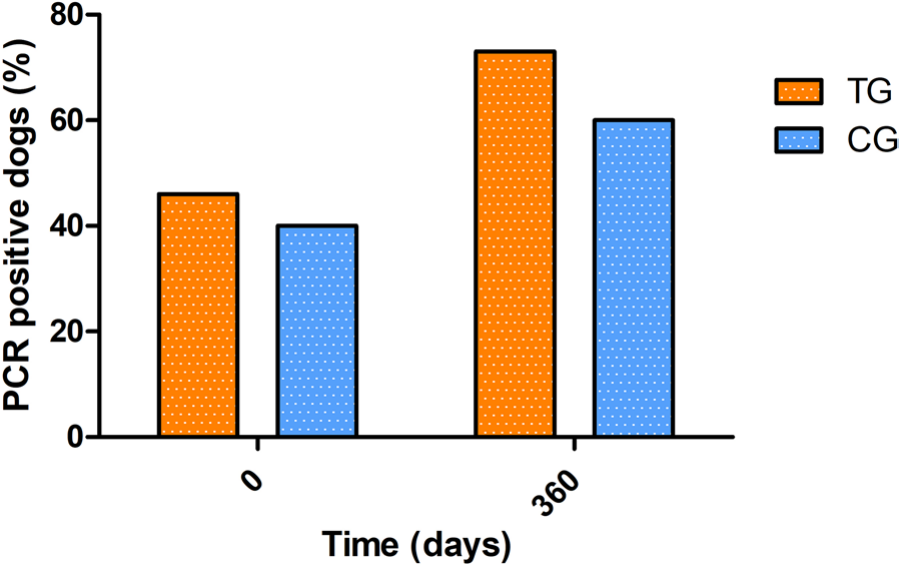
*Leishmania* PCR results of the dogs with disease development at the beginning and end of the study. *CG* control group; *TG* treated group

The *Leishmania* real-time PCR results at the beginning and end of the study of the dogs with disease development and classified by treatment are depicted in Fig. 6. No differences in PCR results in dogs with disease development were detected between treatment groups (*P* > 0.05) (Additional file 5: Table S4).

**Fig. 7:**
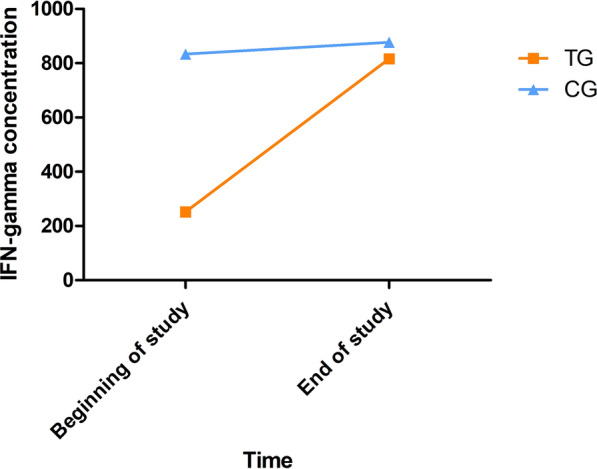
IFN-γ concentrations in the condition of medium with soluble *L. infantum* antigen at the beginning and end of the study. *CG* control group; *TG* treated group

The IFN-γ concentrations in the condition of medium with soluble *L. infantum* antigen at the beginning and end of the study of all dogs classified by treatment are given in Fig. 7. The results of IFN-γ concentrations after stimulation with concanavalin A can be found in Additional file 6: Table S5. An increase of IFN-γ concentration in blood stimulated with soluble *L. infantum* antigen was detected when comparing the results at the beginning and the end of the study of all dogs (Additional file 6: Table S5). No differences in IFN-γ concentration at the beginning and at the end of the study were detected between treatment groups (*P* > 0.05) (Additional file 6: Table S5). When dogs were classified by their initial in-house ELISA result (low positive vs. medium to high seropositive), low-seropositive dogs presented a significantly higher IFN-γ concentration in blood stimulated with soluble *L. infantum* antigen at the beginning and end of the study compared to medium- to high-seropositive dogs (Table 7). No differences in IFN-γ concentration between treatment groups were observed in the low-seropositive dogs or the medium- to high-seropositive dogs (*P* > 0.05). When only dogs that presented an increase of IFN-γ concentration in blood stimulated with soluble *L. infantum* antigen at the end of the study were analyzed, it was detected that low-seropositive dogs treated with domperidone presented a higher increase of IFN-γ production (median = 1831 pg/ml) in blood stimulated with soluble *L. infantum* antigen than those treated with placebo (median = 937.7 pg/ml) (Mann-Whitney *U* test: *U* = 52, *P* = 0.043).

**Table 7:** IFN-γ concentration of seropositive groups at the beginning and end of the study

Conditions	IFN-γ (pg/ml) median (min–max)			
	Low-seropositive dogs (*n* = 48)		Medium- to high-seropositive dogs (*n* = 34)	
	Beginning of the study	End of study	Beginning of the study	End of study
Medium with soluble *L. infantum* antigen	1463^a^ (0–10036)	2018^b^ (0–18969)	16^a^ (0–14190)	90^b^ (0–9546)
Medium with concanavalin A	6929^c^ (1147–43290)	8923^c^ (915–25000)	6748 (860–27880)	7660 (1275–41120)

*IFN-γ* interferon gamma; *max* maximum; *min* minimum

^a^Low-seropositive dogs presented a significantly higher IFN-γ production at the beginning of the study compared to medium- to high-seropositive dogs (Mann-Whitney *U* test: *U*: 450.5, *P* = 0.0006)

^b^Low-seropositive dogs presented a significantly higher IFN-γ production at the end of the study compared to medium- to high-seropositive dogs (Mann-Whitney *U* test: *U*: 435, *P* = 0.0003)

^c^Significantly higher compared to the beginning of the study (Wilcoxon signed-rank test: W: − 406, *P* = 0.038)

**Fig. 8:**
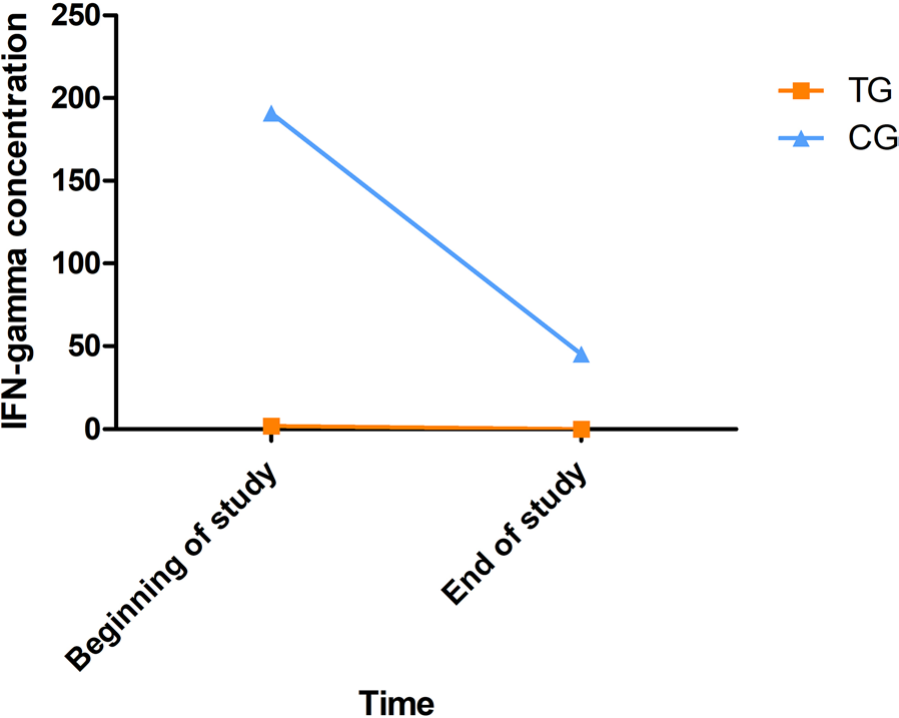
IFN-γ concentrations in the condition of medium with soluble *L. infantum* antigen of dogs with disease development at the beginning and end of the study. *CG* control group; *TG* treated group

The IFN-γ concentrations in the condition of medium with soluble *L. infantum* antigen at the beginning and end the study of the dogs with disease development classified by treatment are depicted in Fig. 8. The results of IFN-γ concentrations after concanavalin A stimulation can be found in Additional file 7: Table S6. No differences in IFN-γ concentration in dogs with disease development were detected between treatment groups (*P* > 0.05) (Additional file 7: Table S6).

### Safety variables

Four dogs in the TG (6%) presented an adverse drug reaction during the study. The four dogs developed self-limiting diarrhea for 1 or 2 days after domperidone administration. This adverse drug reaction only occurred during the first administration of domperidone, which was initiated at day 0. On subsequent administrations starting at days 120 and 240, no adverse drug reaction occurred.

No adverse drug reactions were observed in dogs treated with placebo.

## Discussion

The development of clinical leishmaniosis depends largely on the immune response of the host [7, 8]. Thereby, treatments that can enhance the host's immune system could provide an alternative direction to combating the infection [19, 54, 55]. This is the first published clinical trial testing the clinical efficacy and safety of domperidone (Leisguard®) in healthy dogs seropositive to *L. infantum* infection.

In the present study, it was observed that dogs treated with domperidone were less likely to present disease development than dogs treated with placebo. In addition, dogs treated with placebo presented higher parasitemia at the end of the clinical trial than domperidone-treated dogs, indicating a worse clinical condition. These differences were highly significant in low-seropositive dogs while, in medium- to high-seropositive dogs, disease development was similar in both groups. Furthermore, medium- to high-seropositive dogs also presented higher parasitemia at the end of the study than low-seropositive dogs. This was to be expected as high antibody levels have previously been associated with dissemination of the parasite and clinical disease [5, 41]. Therefore, based on the results of this study, in healthy dogs with high antibody levels, treatment with domperidone alone would not be enough to avoid disease development.

Furthermore, it was also observed that low-seropositive dogs presented a significantly higher IFN-γ production in blood stimulated with soluble *L. infantum* antigen than medium- to high-seropositive dogs. These results agree with previous studies that have reported that IFN-γ producer dogs commonly present lower antibody levels, lower blood parasitemia and milder clinical expression of clinical leishmaniosis than IFN-γ non-producer dogs [51, 56, 57]. Similarly, those dogs that presented disease development in the present study presented similar IFN-γ production to medium- to high-seropositive dogs and could be described as IFN-γ non-producer dogs and thus more likely to develop clinical leishmaniosis [51, 56, 57]. In this study, it was also observed that low-seropositive dogs treated with domperidone presented a significantly higher increase of IFN-γ concentration in blood stimulated with soluble *L. infantum* antigen at the end of the study than those treated with placebo. Domperidone is known to be able to increase blood prolactin levels, which also induces a boost of CD4^+^ T lymphocytes and the release of cytokines such IFN-γ [29, 30]; thus, treatment with domperidone should be able to increase the release of IFN-γ as observed in the present clinical trial. These results could also be compared to those observed in IFN-γ non-producer dogs with clinical leishmaniosis during long-term traditional treatment [51], which, in addition to significant increase of IFN-γ production, also presented a decrease in *L. infantum* antibody levels and blood parasitemia and clinical improvement.

In the present study, most of the dogs that developed disease were classified in LeishVet clinical stage IIa and presented clinicopathological abnormalities such as hyperglobulinemia, hyperproteinemia and decreased A/G ratio while a minority also presented clinical signs such as skin lesions or weight loss. These clinicopathological findings and clinical signs have already been described in dogs with leishmaniosis [5, 52]. The fact that the majority of the dogs only presented clinicopathological abnormalities with no clinical signs, and thus were apparently healthy, highlights the importance of performing routine laboratory tests in apparently healthy *L. infantum*-seropositive dogs to detect disease development and progression, which could shorten treatment duration and also improve disease prognosis [5, 10]. Furthermore, even though renal azotemia has been labeled as an uncommon laboratory finding in apparently healthy dogs [2, 5, 6], one dog presented renal azotemia with proteinuria in this study. In our case, the follow-up was every 4 months, and this dog presented unexpected renal azotemia with proteinuria during this short period of time. These results not only highlight the importance of performing routine laboratory tests in both blood and urine again, but also the importance of a controlled follow-up that should be shorter than 4 months, similar to the monitoring recommended in dogs under treatment for leishmaniosis, which should be every 3 to 4 months [2, 5].

Surprisingly, no differences were found in antibody levels during the follow-up period between dogs treated with domperidone and dogs treated with placebo. Also, few dogs (< 10%) presented seroreversion (were seronegative at some point during the follow-up), although only three dogs were seronegative at the end of the study. As the clinical trial was performed over a year, these results could be affected by the seasonal dynamics of anti-*L. infantum* antibody levels. In previous studies [58, 59], a significant reduction in antibody levels was observed during the non-sandfly transmission season in dogs [58]. However, in the present study, most of the dogs that presented seroreversion did so during sandfly season (Additional file 4). In a previous study [21], a reduction of anti-*L. infantum* antibody levels was observed in dogs with leishmaniosis affected by chronic kidney disease and treated with domperidone. In this previous study [21], a low number of dogs with clinical disease (*n* = 14) were followed for 180 days and showed a significant reduction in serum creatinine, C-reactive protein, globulins and anti-*L. infantum* antibody levels. In the present study, even if no differences were found in antibody levels between different treatments, a statistical increase of antibody levels in those dogs with disease progression was observed at days 120 and 240. These observations were to be expected as the increase of anti-*L. infantum* antibody levels is usually linked to disease progression in dogs with *L. infantum* infection [5, 41].

Additionally, only four dogs treated with domperidone presented mild adverse drug reactions. Those four dogs presented a self-limiting diarrhea for 1 or 2 days. This adverse drug reaction is already listed on the label [60]. Furthermore, it is detailed that this effect should disappear after the treatment is withdrawn [24, 60, 61], although in the present study the treatment with domperidone was not withdrawn, as the treatment was still administered for 4 consecutive weeks, and the diarrhea also disappeared. Therefore, the administration of domperidone in healthy seropositive dogs appears to be safe.

Healthy *L. infantum*-seropositive dogs are usually scientifically neglected, and there is no strong evidence of whether it is better to monitor them without treatment or treat them with conventional anti-*Leishmania* drugs or immunotherapy [2, 5]. For example, a previous study that treated clinically healthy *Leishmania*-infected dogs with dietary nucleotides showed that the use of dietary nucleotides was safe and could be able to reduce the rate of disease progression, although it was also stated that further clinical trials with larger sample sizes and other drug combinations were needed to confirm these observations [62]. In the present clinical trial, domperidone was also able to reduce disease progression, especially in low-seropositive dogs, and thus could be used as treatment for those scientifically neglected dogs that are infected by *L. infantum* but do not present clinical disease [5]. Furthermore, the advantages of domperidone compared to other products are that it is a treatment that can be administered orally and presents a very good safety profile. Moreover, domperidone is already being administered as treatment in healthy *L. infantum*-seropositive dogs in the clinical setting [63–65]. For example, in a questionnaire-based survey performed in Portugal [63], subclinically infected dogs were not treated in > 50% of the cases, but, if a treatment was administered, the first choice protocol was domperidone. Another questionnaire-based survey performed in Spain [64] also reported the use of domperidone (alone or combined with allopurinol) as first- or second-line treatment for CanL.

This clinical trial presents certain limitations that could have affected the outcome of the study. One of the limitations is that the sample size of dogs included in the study (111 dogs) was not the same as the one previously calculated (174 dogs). Sample size estimation is a critical step in planning a clinical trial as it may lead to rejection of an efficacious product, approval of an ineffective product and ethical issues related to product exposition to more subjects than necessary [40]. When a sample size is overestimated (the sample size selected is less than what was calculated), the statistical analysis may result in non-significance, even though clinical significance exists [40]. Significant differences were observed between domperidone and placebo, even though the sample size was lower than calculated; therefore, sample size was likely overestimated in the present study. Furthermore, analysis was performed following the intention-to-treat principle [53] that could affect the results of the statistical analysis. This principle maintains the effect of randomization in the clinical trial as all participant dogs that have been assigned to a group (TG or CG) are included in the statistical analyses even if they did not follow the right protocol or were withdrawn from clinical trial [53, 66]. The intention-to-treat principle is considered a conservative strategy to interpret clinical trials and is less likely to detect a new treatment as effective. The inclusion of all animal’s data could weaken the outcome of the clinical trial [53, 66]. In the present study, even if a conservative data interpretation was used, significant differences were observed between domperidone and placebo. Finally, another limitation of the clinical trial is the absence of an initial screening for other infectious diseases at the time of enrollment of the dogs as co-infections are known to increase the risk of development of clinical leishmaniosis in dogs and could also affect the effectivity of immunotherapy [67, 68].

Further studies must investigate the use of domperidone in dogs with clinical leishmaniosis in combination with traditional therapies such as antimonials and allopurinol. The use of immunotherapy could shorten treatment duration, which could reduce the incidence of adverse effects produced by traditional therapies and also improve the prognosis [12, 13, 16, 19]. Furthermore, studies on the effect of domperidone on transmissibility of *L. infantum* could be an interesting area of future research as seropositive healthy dogs (subclinical dogs) are an important consideration for public health [69, 70].

## Conclusions

This study shows that the use of domperidone in healthy *L. infantum*-seropositive dogs proved to be effective against disease development, especially in dogs with low *L. infantum* antibody levels compared to dogs treated with placebo. Therefore, domperidone appears to be a drug to be used in healthy dogs with low antibody levels in the clinical setting. Furthermore, domperidone presented a good safety profile.

### Supplementary Information

**Additional file 1:** Raw data of the clinical trial.

**Additional file 2: Table S1.** Signalment, immunological and parasitological status, clinical findings and LeishVet clinical staging of dogs with disease development.

**Additional file 3: Table S2.** Endpoint ELISA results of the dogs at days 0, 120, 240 and 360.

**Additional file 4: Table S3.** Number of seronegative dogs at each visit (days 0, 120, 240 and 360) and if they were sampled during sandfly season.

**Additional file 5: Table S4.** *Leishmania* PCR results of the dogs with disease development at the beginning and end of the study.

**Additional file 6: Table S5.** IFN-γ concentration of the dogs at the beginning and end of the study.

**Additional file 7: Table S6.** IFN-γ concentration of dogs with disease development at the beginning and end of the study.

## Data Availability

All raw data of the clinical trial is availabe as supplementary material.

## Abbreviations

A/G: Albumin/globulin
AHCC: Active hexose dietary compound
CanL: Canine leishmaniosis
CBC: Complete blood count
CG: Control group
Ct: Cycle threshold
EU: ELISA units
IFN-γ: Interferon gamma
NK: Natural killer
OD: Optical density
PBS: Phosphate-buffered saline
RT-PCR: Real-time PCR
UPC: Urinary protein creatinine ratio
TG: Treated group
WBA: Whole-blood stimulation assay
